# The Efficacy of Glucagon-like Peptide-1 Based Therapies in Heart Failure Across the Spectrum of Left Ventricular Ejection Fraction: A Systematic Review and Meta-Analysis

**DOI:** 10.3390/jcm15124450

**Published:** 2026-06-09

**Authors:** Theodoros Christophides, Gisella Figlioli, Sotiris Christoforou, Kyriacos Ioannou, Maria Kounnafi, Daniele Piovani, Stefanos Bonovas, Georgios Nikolopoulos

**Affiliations:** 1 Department of Cardiology, Nicosia General Hospital, State Health Services Organisation, Paleos Dromos Lefkosias-Lemesou No. 215, 2029 Strovolos, Nicosia, Cyprus; 2 Medical School, Shacolas Educational Centre for Clinical Medicine, University of Cyprus, Paleos Dromos Lefkosias-Lemesou No.215/6, 2029 Strovolos, Nicosia, Cyprus; christoforou.sotiris@ucy.ac.cy (S.C.); ioannou.kyriakos@ucy.ac.cy (K.I.); kounnafi.maria@ucy.ac.cy (M.K.); 3 Department of Biomedical Sciences, Humanitas University, Via Rita Levi Montalcini 4, 20072 Pieve Emanuele, Milan, Italy; gisella.figlioli@hunimed.eu (G.F.); daniele.piovani@hunimed.eu (D.P.); stefanos.bonovas@hunimed.eu (S.B.); 4 IRCCS Humanitas Research Hospital, Via Alessandro Manzoni 56, 20089 Rozzano, Milan, Italy

**Keywords:** heart failure, GLP-1 receptor agonists, ejection fraction, cardiovascular outcomes, randomised controlled studies

## Abstract

**Background/Objectives:** Despite advances in heart failure (HF) pharmacotherapy, novel treatments are needed for its main subtypes: preserved (HFpEF), mildly reduced (HFmrEF), and reduced (HFrEF) ejection fraction. Glucagon-like peptide-1 (GLP-1) based therapies have shown cardioprotective effects. We conducted a systematic review and meta-analysis (Prospero Registration Number CRD42024606997) assessing the efficacy of GLP-1 based therapies (GLP-1 receptor agonists including tirzepatide) across the spectrum of left ventricular ejection fraction on cardiovascular (CV) outcomes. **Methods**: The PubMed, Embase, Scopus and trial registries were searched until December 2025 for randomised controlled trials (RCTs) involving adults with HF treated with GLP-1 based therapies. Outcomes included heart failure hospitalisations (HFH), CV, and all-cause mortality. Pooled relative risks (RRs) with 95% confidence intervals (CIs) were calculated using random-effects models. Subgroup analyses were performed by HF subtype, age, coronary artery disease (CAD) presence, and GLP-1 based therapeutic agent. **Results**: Fourteen RCTs (15,180 participants), at low risk of bias, were included. These agents significantly reduced HFH (RR: 0.84, 95% CI: 0.71–0.99), especially in HFpEF patients with stable CAD (RR: 0.61, 95% CI: 0.46–0.79). Limited data suggested benefits for exenatide in HFmrEF patients (RR: 0.67, 95% CI: 0.47–0.95). CV mortality was reduced in HFrEF patients <65 years old (RR: 0.71, 95% CI: 0.54–0.95). Benefit was seen in composites of HFH and CV mortality for HFpEF (RR: 0.67, 95% CI: 0.54–0.83). **Conclusions**: GLP-1 based therapies may reduce HFH in HFpEF and possibly lower CV mortality in younger HFrEF patients, suggesting phenotype-specific effects.

## 1. Introduction

Pharmacotherapy remains the cornerstone of heart failure (HF) treatment, and over the past decade, significant advancements have been made in the discovery and study of potential novel treatments, based on an improved understanding of its pathophysiology [1]. With the expected rise in HF incidence, however, there is an increasing need for introducing novel and efficacious treatments, especially for the HF with preserved (HFpEF) and mildly reduced (HFmrEF) ejection fraction subgroups, where there is lack of trial-supported prognostic therapies, with the exception of sodium-glucose co-transporter 2 inhibitors (SGLT2-i) [2].

Recently, the focus has shifted to glucagon-like peptide-1 (GLP-1) based therapies (GLP-1 receptor agonists (GLP-1 RAs) including tirzepatide) and their impact on HF outcomes. Substantial and growing evidence from cardiovascular (CV) outcome trials demonstrates a consistent and significant reduction in atherothrombotic events, particularly among patients with established atherosclerotic CV disease [3,4]. Further to their metabolic effects, the pleiotropic mechanisms of action of GLP-1 based therapies offer additional benefits to the CV system through mechanisms such as promoting natriuresis, lowering blood pressure, improving cardiac contractility, and exerting anti-inflammatory, antioxidative, and anti-atherogenic actions [5]. GLP-1 RAs have been associated with a reduction in hospitalisations for new-onset HF, independent of diabetes status [4]. However, their efficacy in patients with established HF, particularly those with reduced ejection fraction (HFrEF), appears limited or potentially unfavourable [4,6,7]. Meta-analyses support these findings, suggesting that while GLP-1 RAs may lower HF incidence through effects on atherosclerotic disease, they show minimal or no benefit in patients with pre-existing HF [6,8,9]. The complexity increases when considering that most HF cases, especially in patients with obesity and diabetes mellitus (DM), are non-atherosclerotic in aetiology [4]. Additionally, most clinical trials to date were not designed or powered to specifically assess a broad range of HF outcomes or hard end-points, and often lacked adequate representation of distinct HF phenotypes or subgroups, especially in the case of HFmrEF and HFpEF [4].

Given the variability in results, current guidelines remain inconclusive regarding GLP-1 RA use in HF [2]. Thus, there is a need to definitively answer the practical questions as to whether GLP-1 based therapies are indeed a potentially novel treatment for HF which may rival other antidiabetic agents such as the SGLT2-i, and as importantly which HF subgroups benefit the most. To this end, we conducted a systematic review to comprehensively evaluate the effects of GLP-1 based therapies in HF across the spectrum of left ventricular ejection fraction (LVEF) with regards to their potential impact on CV outcomes, including heart failure hospitalisations (HFH) and mortality.

## 2. Materials and Methods

### 2.1. Search Strategy and Study Identification

This review followed the Cochrane Guidance and the Preferred Reporting Items for Systematic Reviews and Meta-Analyses (PRISMA) 2020 guidelines (see also Supplementary Table S1) [10,11]. No institutional review board or ethics committee approval was required or sought as this systematic review relied solely on previously published data and did not involve the collection of new data from human participants. The study protocol was registered with PROSPERO (number CRD42024606997, registered 2024). A thorough search was performed on the PubMed, Embase, and Scopus databases until December 2025, with supplementary searches in the ClinicalTrials.gov register to identify interventional trials reporting on outcomes of interest regarding the use of GLP-1 based therapies in HF patients. A search strategy was generated based on the research question and the PICO framework (P-patient, population, I-intervention, C-comparator, control, O-outcomes), adapted for each database searched. This strategy included: (a) Participants/population: adults (18 years of age and older) of both sexes and any ethnicity with a diagnosis of HF; (b) Intervention: GLP-1 based therapies that have received approval from the American and European regulatory agencies on medicinal products and are in clinical use; (c) Comparator/control: Placebo-control or active control (e.g., insulin, antidiabetic drugs which have known benefits or no adverse outcomes in HF patients, such as SGLT2-i, and lifestyle modifications); (d) Outcomes: see relevant subsection below on outcomes.

Only randomised controlled trials (RCTs) were included. The search was restricted to human studies, but no other limitations such as language or publication date were applied. Search terms included ‘heart failure’, ‘glucagon-like peptide-1 receptor agonist’, ‘GLP-1 receptor agonist’, ‘GLP-1 RA’, ‘exenatide’, ‘albiglutide’, ‘liraglutide’, ‘dulaglutide’, ‘semaglutide’, ‘lixisenatide’, and ‘tirzepatide’. Appropriate Medical Subject Heading terms, truncated terms, and variants were used.

Previous meta-analyses and systematic reviews evaluating GLP-1 based therapies were assessed for retrieving relevant studies. Search results were downloaded and deduplicated using Mendeley software (version 2.127.1, Elsevier Inc., New York, NY, USA, https://www.mendeley.com). They were then transferred to a digital data collection form for further study selection.

### 2.2. Study Selection, Data Extraction, and Risk of Bias

Abstract and full text screening was performed by four independent reviewers (T.C., S.C., K.I. and M.K.), and the decisions made for each article were documented on the study collection form. Any disagreement among reviewers was discussed and resolved by consensus. Subsequently, one reviewer (T.C.) extracted study and patient characteristics, intervention, comparator, and outcome data from the included studies using a separate digital collection form, and three independent reviewers (S.C., K.I., and M.K.) checked the data for accuracy and detail. In cases of disagreements between the reviewers, consensus was reached through discussion with another author (G.N.). Where necessary, data were further extracted from supplementary study material.

The revised Cochrane risk-of-bias tool (RoB 2) was used to assess the risk of bias in the results of the included studies [12]. Five domains were assessed for each of the selected trials, relating to the study design/conduct (randomisation, blinding, allocation), and reporting on outcomes.

### 2.3. Data Collection: Eligible Trial Design, Participant Characteristics, and Outcomes

For each eligible trial, the data collected regarding its design included: year of publication, study duration, participant centres/countries, presence of blinding, GLP-1 therapeutic agent used, target HF subtype, follow-up duration, type of control, number of participants in the exposed and control groups and analysis type. With regards to baseline participant characteristics, these included: male/female proportions, age of participants, body mass index (BMI) status, presence of DM, presence of coronary artery disease (CAD), New York Heart Association (NYHA) status, pre-treatment with prognostic HF drugs and LVEF status. Data were collected for three primary outcomes (with associated composites). These included: time to first worsening of HF events or HFH (defined as hospitalisation or urgent clinical visit for acute deterioration of HF with symptoms and signs), time to all-cause mortality, time to CV death, composite of first worsening of HF events and all-cause mortality, composite of first worsening of HF events and CV death.

### 2.4. Data Analysis

A meta-analysis was performed to assess potential benefits and harms associated with the use of GLP-1 based therapies (compared with placebo or active control) in patients with HF. The random effects model (DerSimonian–Laird approach) was used to pool the effect estimates of the individual studies. Hazard ratios (HRs) were primarily used to estimate relative risk (RRs). When HRs were not available (very few occasions), risk ratios were used instead. For zero counts, 0.5 was added to each cell.

Statistical heterogeneity was investigated with the Cochran’s Q test, with a 0.10 level of significance, and quantified using the I^2^ statistic, which reports the proportion of variability across studies that is due to between-study heterogeneity rather than chance. The I^2^ statistic was interpreted according to the Cochrane Handbook’s guide: 0–40% might not be important; 30–60% may represent moderate heterogeneity; 50–90% may represent substantial heterogeneity; while 75–100% represents considerable heterogeneity [11]. Publication bias was assessed using funnel plots (for outcomes evaluated by ten or more studies), the Begg’s adjusted rank correlation test, and the Egger’s regression asymmetry test. Restricted maximum likelihood (REML) was used to estimate the between-study variance in the funnel plots. Subgroup analysis was performed depending on data availability, and included HF subtypes, LVEF status, GLP-1 based therapeutic agent, age and sex differences, presence of DM and/or CAD, BMI class, and pre-treatment with HF prognostic drugs. A Cochran’s Q statistic (Q_b_) was used to test differences between subgroups. Under the null hypothesis of homogeneity across the subgroups, Q_b_ follows a chi-squared distribution with L-1 degrees of freedom, where L is the number of subgroups. No participant population was represented more than once within any pooled analysis. Consequently, statistical adjustment for overlapping populations was not necessary.

All of the analyses were performed using Stata software (versions 18 and 19.5, StataCorp, College Station, TX, USA). A two-tailed *p*-value < 0.05 was considered statistically significant for all analyses, except for the assessment of heterogeneity (where a level of 0.10 was used).

## 3. Results

### 3.1. Literature Search and Study Selection

**Figure 1:**
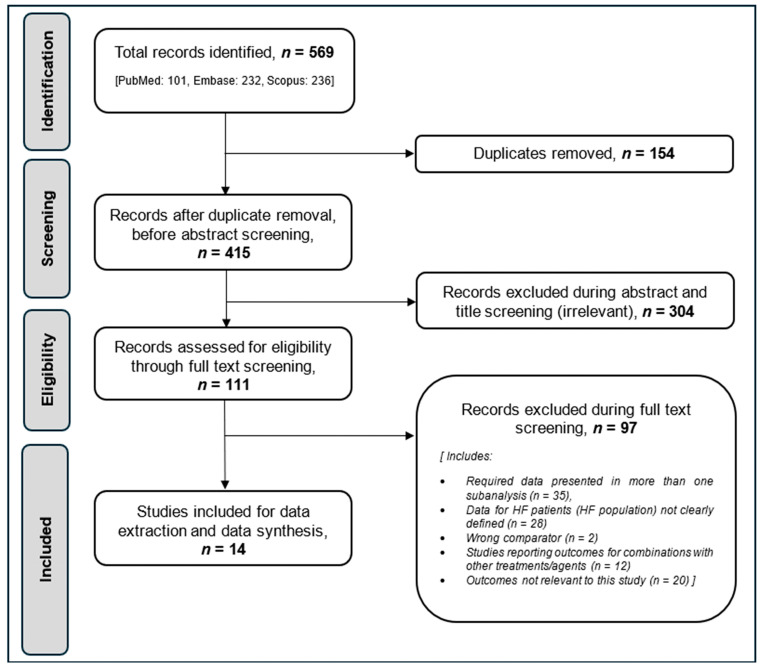
PRISMA flowchart for study selection. Abbreviations: HF, heart failure.

The PRISMA flowchart in Figure 1 shows the selection process and reasons for exclusion at each stage. In total, 569 records were identified during the literature search using pre-specified algorithms. Of these, 154 were removed as duplicates. A further 401 were excluded after title, abstract, and full-text screening, leaving 14 studies for meta-analysis. The studies consisted of five trials assessing GLP-1 based therapies in HF patients (analysed on an intention-to-treat (ITT) basis) [7,13,14,15,16], and nine subgroup analyses of existing trial data, assessing a broad range of cardiovascular outcomes [17,18,19,20,21,22,23,24,25].

### 3.2. Assessment of Bias

The risk of bias assessment was performed, with results shown in Supplementary Figure S1 (generated using the Robvis software application) [26]. Overall, risk of bias was low, though concerns arose in three studies regarding allocation concealment, participant randomisation, and assessor blinding, mainly due to insufficient detail about these processes [13,15,18]. Outcomes were reported appropriately across studies.

Assessment for publication bias (Supplementary Figure S2) was negative in analyses for HFH (Begg’s test, *p* = 0.38; Egger’s test, *p* = 0.25). As fewer than ten studies were included in analyses of the remaining outcomes, formal publication bias assessment was not deemed appropriate.

### 3.3. Study and Patient Characteristics

The study designs and participant characteristics are shown in Supplementary Table S2. Most studies were multicentre and multinational (*n* = 11, 79%). All were randomised, double-blinded, and placebo controlled. The studies were conducted between 2009 and 2024, with the first relevant publication in 2015, and involved 15,180 participants in total. Diabetics were included in all but two of the studies [21,24]. Patients with CAD were included in 12 studies (in three studies patients had acute coronary syndromes), whereas two studies provided no specific details on ischaemic heart disease patients [18,25]. Participant BMI was defined in most studies, with values mainly in the obese range (BMI ≥ 30). Seven GLP-1 based therapeutic agents were assessed (albiglutide, dulaglutide, exenatide, liraglutide, lixisenatide, semaglutide, and tirzepatide), with most studies including semaglutide (*n* = 5, 36%).

### 3.4. Study Outcomes—Heart Failure Hospitalisations

The overall population comprised 8914 participants, with a mean age of 64.5 years (standard deviation: ±2.76 years). Approximately 65% of the study population were male. The mean follow-up duration across studies was 2.8 years. A pooled analysis of ten studies (14 RRs as two studies provided separate estimates for HF phenotypes) [22,23] showed a significant reduction in the risk of HFH (RR: 0.84, 95% confidence interval (CI): 0.71–0.99) [7,13,15,16,17,18,19,20,22,23]. The analysis showed moderate heterogeneity (Cochran’s Q test, *p* = 0.02; I^2^ = 49.5%).

**Figure 2:**
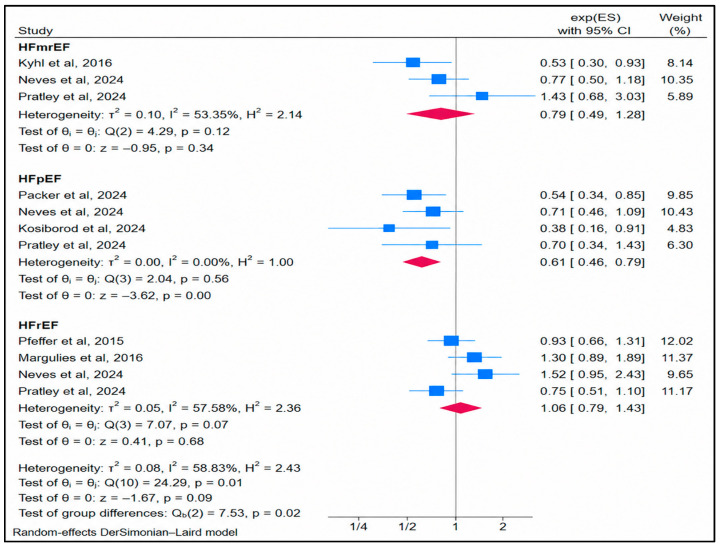
Forest plot of the pooled effect of GLP-1 based treatments on heart failure hospitalisations, according to heart failure subgroup. Abbreviations: CI, confidence interval; exp(ES), exponentiated effect size (i.e., relative risk); HFmrEF, heart failure with mildly reduced ejection fraction; HFpEF, heart failure with preserved ejection fraction; HFrEF, heart failure with reduced ejection fraction [7,13,15,16,17,22,23].

A pre-specified subgroup meta-analysis found that the effect varied significantly by HF subtype (Q_b_ test of HF group differences, *p* = 0.02) (Figure 2). For patients with HFpEF and stable CAD, there was a clear reduction in HFH (RR: 0.61, 95% CI: 0.46–0.79), with no evidence of statistical heterogeneity (Cochran’s Q test, *p* = 0.56; I^2^ = 0%) [15,16,22,23]. Excluding the study that provided data for calculating a risk ratio rather than reporting an HR did not change the results [16]. This observation was consistent when assessing absolute values for LVEF, with a benefit in patients with LVEF > 40% (RR: 0.67, 95% CI: 0.53–0.85; Cochran’s Q test, *p* = 0.27; I^2^ = 21%) [15,16,17,22,23]. Results were neutral in patients with HFrEF and HFmrEF. There was insufficient data to draw valid inferences for patients presenting with acute coronary syndromes (ACS).

A clear benefit was also observed in patients with HFpEF aged ≥65 years (RR: 0.54, 95% CI: 0.38–0.77; Cochran’s Q test, *p* = 0.57; I^2^ = 0%) [15,16,23]. The data was insufficient to assess younger patients. Evidence from two studies suggested potential harm in those with HFrEF, but this was based on limited data (RR: 1.38, 95% CI: 1.03–1.85; Cochran’s Q test, *p* = 0.61; I^2^ = 0%; Q_b_ test of HF group differences, *p* = 0.01) [7,22].

From pooled analyses of a limited number of studies, semaglutide demonstrated significant benefit in patients with HFpEF (*n* = 2 studies; RR: 0.54, 95% CI: 0.30–0.98; Cochran’s Q test, *p* = 0.29; I^2^ = 12%), and exenatide in those with HFmrEF (*n* = 2 studies; RR: 0.67, 95% CI: 0.47–0.95; Cochran’s Q test, *p* = 0.30; I^2^ = 5.9%) [16,17,22,23]. Tirzepatide, a glucose-dependent insulinotropic polypeptide (GIP) analogue and a GLP-1 RA (dual action), was used in one study showing a benefit in patients with HFpEF (RR: 0.54, 95% CI: 0.34–0.85) [15]. In the context of sensitivity analysis, excluding this study, which employed a dual GIP analogue/GLP-1 RA, did not change the results for patients with HFpEF (*n* = 3 studies; RR: 0.64, 95% CI: 0.46–0.90; Cochran’s Q test, *p* = 0.43; I^2^ = 0%) [16,22,23]. No conclusive inferences could be made for HFrEF or other GLP-1 RAs owing to inadequate data.

Finally, restricting the meta-analysis to the 4 ITT primary trials for the effect of GLP-1 based therapies on HFH yielded similar results for all HF phenotypes (for example, in two studies assessing HFpEF; RR: 0.50, 95% CI: 0.33–0.75; Cochran’s Q test, *p* = 0.48; I^2^ = 0%) [7,13,15,16].

### 3.5. Study Outcomes—Mortality (All-Cause and Cardiovascular)

**Figure 3:**
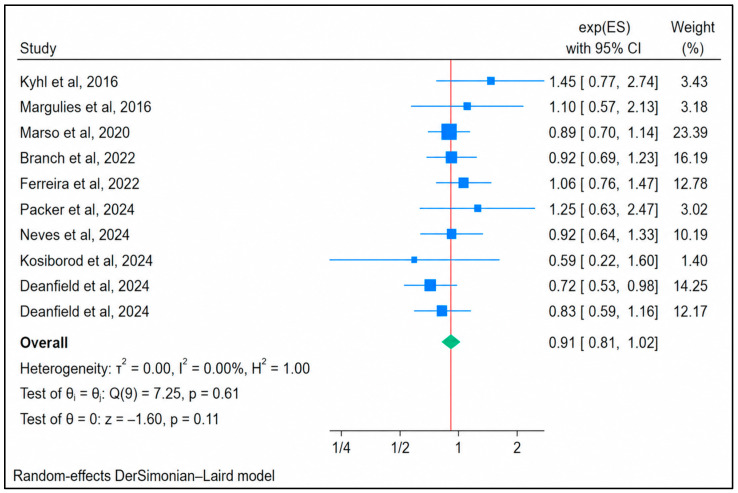
Forest plot of the association between GLP-1 based treatments and all-cause mortality. Abbreviations: CI, confidence interval; exp(ES), exponentiated effect size (i.e., relative risk) [7,15,16,17,18,19,20,22,24].

A pooled analysis (Figure 3) of nine studies (ten RRs, as one study provided separate estimates for HF phenotypes) [24] demonstrated neutral effects on all-cause mortality (RR: 0.91, 95% CI: 0.81–1.02; Cochran’s Q test, *p* = 0.61; I^2^ = 0%) [7,15,16,17,18,19,20,22,24]. The dataset included 10,498 participants (mean age 63.8 ± 2.6 years), of whom 67% were male, with a mean follow-up of 3.0 years. Results were consistent across subgroups stratified by LVEF (including further stratification by type of RCT, i.e., ITT and post-hoc subgroup analyses), age, CAD status, and use of the dual GIP analogue/GLP-1 RA tirzepatide. Excluding the study that provided data for calculating a risk ratio rather than reporting a HR did not change the results [16].

**Figure 4:**
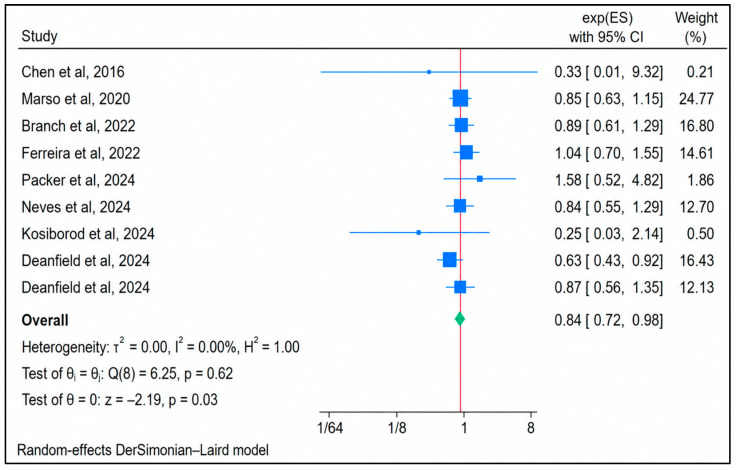
Forest plot of the association between GLP-1 based treatments and cardiovascular mortality. Abbreviations: CI, confidence interval; exp(ES), exponentiated effect size (i.e., relative risk) [14,15,16,18,19,20,22,24].

An analysis (Figure 4) of eight studies (nine RRs as one study provided separate estimates for HF phenotypes) [24] demonstrated a borderline protective effect of GLP-1 RAs on CV mortality (RR: 0.84, 95% CI: 0.72–0.98; Cochran’s Q test, *p* = 0.62; I^2^ = 0%) [14,15,16,18,19,20,22,24]. This dataset included 9954 participants (mean age 64.0 ± 2.7 years), 65% male, with mean follow-up of 3.0 years. Estimates suggested a significant benefit in HFrEF (2 studies involving post-hoc analyses in patients <65 years) [22,24], (RR: 0.71, 95% CI: 0.54–0.95; Cochran’s Q test, *p* = 0.32; I^2^ = 0%). However, the Q_b_ test of HF group differences did not show significant variation across HF subtypes (*p* = 0.56). Exclusion of the study that used tirzepatide did not change the overall pattern of the results [15]. The data was insufficient to draw conclusions regarding mortality (all-cause and CV) for HFmrEF or individual GLP-1 based agents. Exclusion of the two studies that provided data for calculating a risk ratio rather than reporting an HR did not alter the results [14,16].

### 3.6. Study Outcomes—Composite Heart Failure Hospitalisations and Mortality

**Figure 5:**
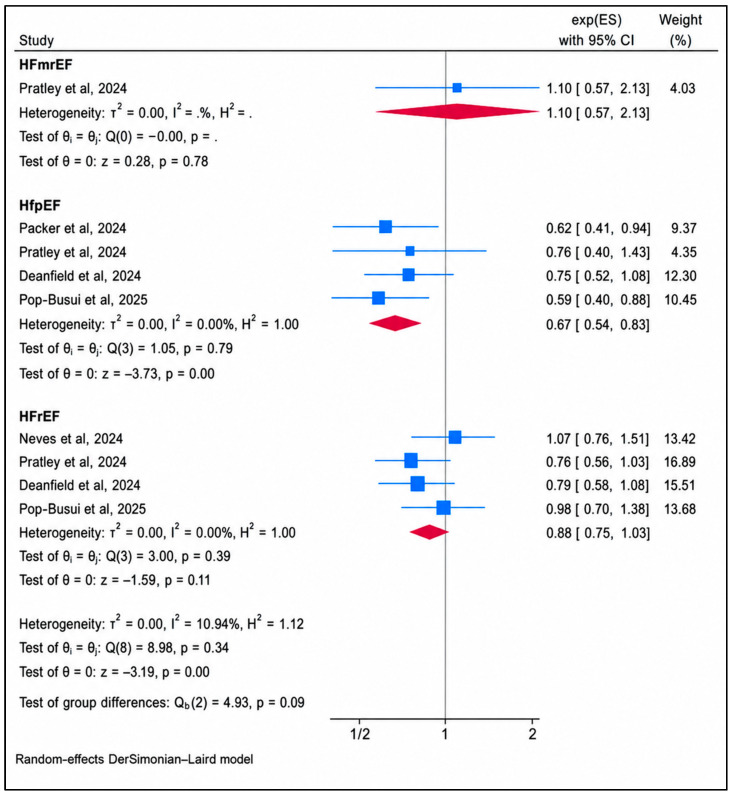
Forest plot of the pooled effect of GLP-1 based treatments on the composite of heart failure hospitalisations and cardiovascular mortality, according to heart failure subgroup. Abbreviations: CI, confidence interval; exp(ES), exponentiated effect size (i.e., relative risk); HFmrEF, heart failure with mildly reduced ejection fraction; HFpEF, heart failure with preserved ejection fraction; HFrEF, heart failure with reduced ejection fraction [15,22,23,24,25].

In terms of the composite outcome of HF hospitalisations and all-cause mortality, the meta-analysis included only four studies, yielding a non-significant RR (RR: 1.01, 95% CI: 0.85–1.21; Cochran’s Q test, *p* = 0.27; I^2^ = 23.16%) [7,17,18,22]. The analysis of the composite outcome of HF hospitalisations and CV-related mortality included eight studies (12 RRs, as three studies provided separate estimates for HF phenotypes) [23,24,25] and did not demonstrate significant effects, except for HFpEF, where GLP-1 based therapeutic agents reduced HFH and CV mortality compared with controls (RR: 0.67, 95% CI: 0.54–0.83; Cochran’s Q test, *p* = 0.79; I^2^ = 0%) (Figure 5) [15,18,19,20,22,23,24,25]. The difference between the HF phenotypes was not statistically significant at the 5% level (Q_b_ test of HF group differences, *p* = 0.09).

For semaglutide, pooled data from three studies, including patients with HFrEF, suggested a marginally significant benefit for HFH and CV mortality (RR: 0.83, 95% CI: 0.69–1.00; Cochran’s Q test, *p* = 0.51; I^2^ = 0%) [23,24,25]. Results for semaglutide in the HFpEF subgroup showed a statistically significant benefit (RR: 0.69, 95% CI: 0.54–0.88; Cochran’s Q test, *p* = 0.64; I^2^ = 0%) [23,24]. Tirzepatide, assessed in one study, demonstrated benefits in patients with HFpEF (RR: 0.62, 95% CI: 0.41–0.94) [15]. Excluding this study from the analysis had no impact on the positive effect of GLP-1 RAs that has been observed in patients with HFpEF (*n* = 3 studies with semaglutide as described above; RR: 0.69, 95% CI: 0.54–0.88; Cochran’s Q test, *p* = 0.64; I^2^ = 0%) [23,24,25]. The above-mentioned study that showed the protective role of tirzepatide in the composite outcome of HF hospitalisations and CV-associated mortality in HFpEF was the only one with an ITT design in this meta-analysis [15]. The remaining estimates were the product of subgroup analyses in RCTs [23,24,25].

## 4. Discussion

In our study, we evaluated the effects of GLP-1 based therapies (GLP-1 RAs including tirzepatide) in patients with rigorously characterised HF, stratified by LVEF and HF subgroups (an approach not commonly undertaken by previous studies in this field). This stratification allowed us to examine the impact of these agents on hard outcomes such as mortality and HFH within all subgroups of HF, thereby emphasising that these therapies are not universally applicable to all HF patients but should be targeted based on HF phenotype. Our meta-analysis suggests that GLP-1 based therapies may be associated with a reduced risk of HFH in patients with HFpEF, with the greatest potential benefit observed among older adults. There is also evidence that these agents may provide clinical advantages in patients with HFmrEF. However, these observations should be considered hypothesis-generating, given the limited number of studies. Furthermore, our findings suggest a potential association between GLP-1 based therapy and reduced CV mortality among younger individuals with HFrEF, although this remains exploratory and needs validating with prospective studies. The above observations suggest that the therapeutic benefits could vary across different HF phenotypes and age groups. Clinically, this highlights the need for tailored therapeutic strategies, supporting more individualised use of these agents in HF management.

The most robust evidence for GLP-1 based therapies pertains to patients with HFpEF. Our data indicate a reduction in HFH, particularly consistent in patients aged ≥65 years with stable CAD. These findings are drawn from approximately 9000 participants, the majority of whom had diabetes and a BMI > 30. They align with results from the recent SUMMIT and STEP-HFpEF trials, as well as subgroup analyses from the FLOW and EXCSEL studies, which demonstrated symptom improvement and reduced risk of HF decompensation in patients receiving these agents compared to placebo [15,22,23,27].

The current analysis lacks sufficient data to determine the comparative efficacy of individual agents within this subgroup, and thus it remains unclear whether the observed benefits represent a class effect or are specific to certain agents. Nonetheless, semaglutide shows promising results, consistent with existing literature [27,28]. Of note, with regards to pre-treatment with SGLT2-i, in the studies analysed here there was minor usage amongst participants, thereby reinforcing the potential independent effect of GLP-1 based therapies in patients with HFpEF.

HFpEF is the predominant form of HF in older adults, particularly those over the age of 65, and is frequently associated with cardiometabolic comorbidities such as obesity and diabetes, which share common pathophysiological pathways contributing to the development of this condition [29]. Hence, these findings are not surprising. There are several plausible explanations as to why this is observed and which may support the use of GLP-1 based treatments in this subgroup of patients. As explained previously, these agents may benefit HF patients with obesity or diabetes by improving metabolic profiles, reducing inflammation, promoting weight loss, enhancing vascular function, and potentially exerting direct cardioprotective effects [5]. These mechanisms are particularly relevant in HFpEF, which is often associated with metabolic syndrome and systemic inflammation.

Large trials such as LEADER and SUSTAIN-6 have shown reduced rates of major adverse CV events in patients with type 2 diabetes and established CV disease. The pooled results in our study further strengthen the potential benefit in patients with stable CAD [18,30]. Among contributing mechanisms, GLP-1 RAs are known to improve endothelial function and reduce oxidative stress, and they may stabilise atherosclerotic plaques, all of which are beneficial in patients with established CAD [5].

When evaluating the mortality benefits (CV and all-cause) of GLP-1 based treatments in HFpEF patients, the effects are less definitive than for HFH, although composite outcomes suggest a potential reduction in HFH and CV mortality. There are several explanations for the lack of significant observations. Firstly, the mean study follow-up was approximately 3 years; hence, the trial duration may have been too short to establish long-term benefits. Moreover, in populations with multiple comorbidities, such as elderly patients, improvements in CV outcomes may not necessarily translate into reductions in all-cause mortality, as deaths from non-cardiovascular causes remain prevalent. This is particularly relevant in conditions like HFpEF, which commonly affects older adults and is frequently accompanied by comorbidities, such as diabetes, that contribute to non-cardiovascular mortality.

Only a small number of studies including participants with HFmrEF met the eligibility criteria, limiting inferences for this subgroup. However, limited evidence from two studies suggests that exenatide may be associated with a reduced risk of HFH in these patients [17,22]. The low representation of this HF subgroup may be expected, because this intermediate HF phenotype has been defined more recently, hence data from this group were either omitted or pooled together with those from patients with HFpEF (i.e., as a group with LVEF > 40%) in the majority of the available studies.

Regarding the pathophysiological basis to the above observation with exenatide, HFmrEF is believed to share features of both HFrEF (neurohormonal activation) and HFpEF (metabolic and inflammatory drivers) [2,31]. Exenatide’s metabolic effects, weight loss, and potential for hemodynamic improvement may offer dual benefit in this intermediate phenotype. Furthermore, this HF subgroup tends to be dominated by young males with CAD [2,31]. Hence it is not surprising that in the small study by Kyhl and colleagues in predominantly male patients presenting with an ACS, and which includes observations that fit within this LVEF range, there was a reduction in HF admissions [17].

In contrast to the findings for patients with HFpEF, the results from this meta-analysis have been inconclusive when it comes to patients with HFrEF and HFH. Furthermore, the findings related to mortality outcomes present a nuanced picture. While a reduction in all-cause mortality was observed, the results did not reach statistical significance, suggesting a potential but inconclusive survival benefit. However, a more pronounced effect was noted in CV mortality. This benefit appeared to be most evident in patients under the age of 65 with stable CAD, indicating that age and underlying ischaemic burden may modulate the therapeutic response in this population. These results may contradict the findings of a recent meta-analysis by Merza and colleagues with regards to these outcomes [6]. Nonetheless, as no statistically significant reduction in overall mortality (independent of age) was observed in this subgroup, and given the limited dataset, caution is warranted when generalising these findings to wider clinical practice. Further studies are required to substantiate these observations. GLP-1 RAs may reduce mortality in HFrEF through a multifactorial set of mechanisms, including improvements in metabolic control, cardiac and vascular function, weight loss, and anti-inflammatory effects [5]. These benefits may be particularly relevant to patients with coexisting diabetes or obesity, though the degree of benefit in HFrEF can vary depending on disease severity and individual clinical phenotype. Furthermore, similar to the findings in HFpEF and supporting the concept that age may play a role in the outcome, the potential beneficial effect on younger patients may be of clinical relevance, as patients with HFrEF are often younger than those with preserved LVEF [2].

The weight-reducing effects of GLP-1 based treatments remain a subject of ongoing debate, given their potential to influence HF outcomes. However, the relationship is complex and must be interpreted in the context of the “obesity paradox” seen in HFpEF and HFrEF patients [32]. Although obesity is a well-known risk factor for the development of HF, observational studies in patients with established HF have paradoxically shown an association between higher BMI and improved survival [32]. This phenomenon may reflect reverse causation, the limitations of BMI as a surrogate for body composition, or the potential protective role of a greater metabolic reserve, rather than a true protective effect of excess adiposity [33]. Importantly, unintentional weight loss in HF (often reflecting cachexia or advanced disease) is consistently associated with adverse outcomes, whereas the impact of intentional weight reduction through structured interventions or pharmacotherapy remains incompletely defined [33,34]. Emerging data, particularly in obesity-related HFpEF, suggest that GLP-1 RA-associated weight loss may improve symptoms and functional capacity. However, the relative contributions of weight-dependent versus direct cardiometabolic effects, as well as effects on hard clinical endpoints across HF phenotypes, remain areas of ongoing investigation [33]. Of note, more recently, findings from the SUMMIT trial have demonstrated that patients with HFpEF derived significant cardiovascular benefit even in the absence of marked weight reduction [35]. These observations challenge the notion that the therapeutic effects of GLP-1 based therapies are mediated exclusively through weight loss, suggesting that attributing their cardiovascular benefit solely to weight reduction represents an oversimplification.

Background HF therapy is an important determinant of outcomes and should be considered when interpreting treatment effects [2]. The relatively low or inconsistent use of guideline-directed medical therapies observed in some of the included studies (refer to Table S2 for details) may reflect historical timing. Specific prognostic agents (such as SGLG2-i or angiotensin receptor-neprilysin inhibitors (ARNI)), as well as broader optimisation of quadruple therapy (beta-blockers, mineralocorticoid receptor antagonists (MRAs), renin–angiotensin system inhibition, and SGLT2-i) did not exist or were not indicated for use in HF management. Thus, several of the studies included in our analysis began recruitment before these therapies were licensed, incorporated into clinical guidelines, or widely adopted in routine practice. Even among more established therapies such as beta-blockers, MRAs, or loop diuretics, prescribing patterns and target-dose attainment were variably reported, thus limiting direct comparisons with contemporary standards of optimised HF care.

In addition to the above, some therapies (including ARNI and certain SGLT2-i) are primarily indicated for specific HF subgroups such as HFrEF. As several trials enrolled heterogeneous HF populations or were not designed to target these subgroups, variability in background therapy use was expected. Differences in trial-era standards of care, HF phenotype distribution, and incomplete reporting of background medication intensity or dose optimisation may therefore have influenced observed treatment effects and should be considered when interpreting both efficacy and safety outcomes. In particular, the incremental benefit of GLP-1 based therapeutic agents may differ depending on whether they are evaluated as adjunctive therapy on top of fully optimised contemporary HF treatment versus in historical cohorts receiving less comprehensive neurohormonal blockade. Nonetheless, contemporary HF management increasingly emphasises the synergistic and additive benefits of combined prognostic therapies, and evidence from cardiovascular outcome trials suggests that combinations of cardiometabolic agents (for example, SGLT2-i together with GLP-1 RAs) provide incremental benefit in cardiovascular health [36]. This evolving therapeutic landscape underscores the importance of contextualising older trial data, as the treatment effects observed in earlier studies may not fully reflect the interaction profile or incremental value of GLP-1 based therapies when layered onto modern, guideline-directed, multidrug regimens. Future research should prioritise targeted trials evaluating different combinations of established HF prognostic therapies with these agents to better define the incremental and potentially synergistic effects of these multidrug strategies and to clarify their optimal use across different HF phenotypes.

Considering the strengths of our study, a major advantage lies in the comprehensive strategy used to search across several trial databases, ensuring a broad and unbiased capture of relevant literature. Furthermore, this study aimed to assess a wide range of clinically meaningful outcomes, which were strictly limited to participants with clearly defined HF (with the majority classified into specific subgroups or having documented LVEF values). This is in contrast with the pooled results from patients with a poorly defined diagnosis of HF or no classification of its subtypes amongst participants. This important fact, which was observed in previous pre-specified analyses of original trial data as well as meta-analyses in this field, may have obscured the true effects of GLP-1 based treatments. With regard to the quality of the studies included, the overall risk of bias was generally low on detailed assessment, further reinforcing the credibility of the so-far conclusions. It should, however, be noted that because this review was intended to provide an exploratory, hypothesis-generating synthesis of the available evidence, a structured assessment of evidence was considered beyond its scope and was therefore not performed. Nevertheless, we acknowledge that factors such as indirectness, imprecision, and between-study heterogeneity may influence the overall certainty of the evidence. Formal certainty-of-evidence assessments may be valuable in future reviews and guidelines development as the evidence base continues to mature. Finally, every effort was made to avoid potential duplication of trial data by carefully distinguishing between original trial results and any relevant pre-specified or post-hoc analyses derived from the same dataset.

A key limitation of our analysis is that we pooled data from different GLP-1 based therapies, including both GLP-1 RAs and the dual GIP analogue/GLP-1 RA tirzepatide. These agents differ in pharmacologic profile and magnitude of weight reduction, which may introduce clinical heterogeneity. Although no meaningful discrepancies in direction of effect were observed across agents in the available data, most individual drugs were represented by a limited number of studies, precluding adequately powered, agent-specific analyses. Consequently, our conclusions apply to GLP-1 based therapies as a class rather than to any single compound, and future studies with sufficient data for individual agents are needed to clarify potential differences in efficacy and underlying mechanisms. Furthermore, our analysis combines data from dedicated HF trials, with post-hoc and subgroup analyses derived from cardiovascular outcome trials. This is an important limitation, given the fact that HF phenotypes were not always uniformly defined across studies. It may have affected cross-trial comparability, which may have introduced heterogeneity in our study design. Nonetheless, it should also be noted that Cochran’s Q test and Inconsistency Index showed relatively low variation in most results. Variability and incomplete reporting of background HF therapies across trials, which have been discussed above, represent additional limitations. Several studies were conducted before the widespread adoption of contemporary guideline-directed medical therapy, and information regarding medication intensity and dose optimisation was inconsistently reported. Consequently, the observed treatment effects of GLP-1 based therapies may not fully reflect their incremental benefit when added to modern HF treatment regimens. Finally, variations in reported outcomes were influenced by differences in the metrics used across studies.

## 5. Conclusions

In the era of precision cardiology, and given the heterogeneity of HF as a syndrome, our findings suggest that GLP-1 based therapies (GLP-1 RAs and tirzepatide) may have differential effects across specific HF phenotypes and should potentially be targeted to specific patient subgroups rather than applied universally across all HF populations. However, these findings should be interpreted cautiously given the limited data available for some subgroup analyses, the absence of consistently significant interaction tests, and variability in the reporting of background heart failure therapies across the included trials. As such, our results should be considered hypothesis-generating and may contribute to future research aimed at clarifying whether a more phenotype-specific approach to the use of these agents in HF management is warranted.

## Data Availability

The data extracted from the included studies, along with the analysis code, are available from the corresponding authors upon request.

## Supplementary Materials

The following supporting information can be downloaded at: https://www.mdpi.com/article/10.3390/jcm15124450/s1, Figure S1: Risk of bias assessment for all included studies using the revised Cochrane assessment tool. (A) traffic light plot for individual studies and (B) summary plot [7,13,14,15,16,17,18,19,20,21,22,23,24,25]; Figure S2: Publication bias assessment—funnel plot for heart failure hospitalisations [7,13,15,16,17,18,19,20,22,23]; Table S1: PRISMA checklist [37]; Table S2: Characteristics of studies used in the meta-analysis [7,13,14,15,16,17,18,19,20,21,22,23,24,25].

## Abbreviations

The following abbreviations are used in this manuscript:

ACS	Acute coronary syndromes
ARNI	Angiotensin receptor-neprilysin inhibitors
BMI	Body mass index
CAD	Coronary artery disease
CI(s)	Confidence interval(s)
CV	Cardiovascular
DM	Diabetes mellitus
exp(ES)	Exponentiated effect size
GIP	Glucose-dependent insulinotropic polypeptide
GLP-1	Glucagon-like peptide-1
GLP-1 RA(s)	Glucagon-like peptide-1 receptor agonist(s)
HF	Heart failure
HFH	Heart failure hospitalisations
HFmrEF	Heart failure with mildly reduced ejection fraction
HFpEF	Heart failure with preserved ejection fraction
HFrEF	Heart failure with reduced ejection fraction
HR(s)	Hazard ratio(s)
ITT	Intention-to-treat
LVEF	Left ventricular ejection fraction
MRA(s)	Mineralocorticoid Receptor Antagonist(s)
NYHA	New York Heart Association
PRISMA	Preferred Reporting Items for Systematic Reviews and Meta-Analyses
RCT(s)	Randomised controlled trial(s)
REML	Restricted maximum likelihood
RR(s)	Relative risk(s)
SGLT2-i	Sodium-glucose co-transporter 2 inhibitors
