# Supplementary material for: The Efficacy of Glucagon-like Peptide-1 Based Therapies in Heart Failure Across the Spectrum of Left Ventricular Ejection Fraction: A Systematic Review and Meta-Analysis
Source: J Clin Med. 2026 Jun 9;15(12):4450. doi: 10.3390/jcm15124450 (PMC13301924; doi:10.3390/jcm15124450)
Supplement: Supplementary file 1 [file jcm-15-04450-s001.zip › jcm-4328287-supplementary.pdf]

PRISMA 2020 Checklist - The efficacy of glucagon-like peptide-1 based therapies in heart failure across the spectrum of left ventricular ejection fraction: a systematic review and meta-analysis *(Christophides T, et al)*

Table S1. PRISMA checklist [37]

Section and Topic	Item #	Checklist item	Location where item is reported
Section and Topic			Item #
Title	1	Identify the report as a systematic review.	**Title Page** (page 1)<br>**Abstract** (page 1)<br>**Section 1. Introduction** (page 2)<br>**Subsection 2.1. Search strategy and study identification (in Section 2. Materials and Methods)** (page 3)
**ABSTRACT**			
Abstract	2	See the PRISMA 2020 for Abstracts checklist.	**Abstract** (page 1)
**INTRODUCTION**			
Rationale	3	Describe the rationale for the review in the context of existing knowledge.	**Abstract** (page 1)<br>**Section 1. Introduction** (page 2)
Objectives	4	Provide an explicit statement of the objective(s) or question(s) the review addresses.	**Abstract** (page 1)<br>**Section 1. Introduction** (page 2)<br>**Subsection 2.3. Data collection: eligible trial design, participant**

Table S1. PRISMA checklist			
**Section and Topic**	**Item #**	**Checklist item**	**Location where item is reported**
			**characteristics and outcomes (in Section 2. Materials and Methods)** (page 4)
**METHODS**			
Eligibility criteria	5	Specify the inclusion and exclusion criteria for the review and how studies were grouped for the syntheses.	**Section 2. Materials and Methods (and subsections 2.1–2.3*)*** (pages 3–4)
Information sources	6	Specify all databases, registers, websites, organisations, reference lists and other sources searched or consulted to identify studies. Specify the date when each source was last searched or consulted.	**Abstract** (page 1) **Subsection 2.1. Search strategy and study identification (in Section 2. Materials and Methods)** (page 3)
Search strategy	7	Present the full search strategies for all databases, registers and websites, including any filters and limits used.	**Subsection 2.1. Search strategy and study identification (in Section 2. Materials and Methods)** (page 3) *(Note: The detailed search codes are available from the corresponding authors upon request)*

Table S1. PRISMA checklist

Section and Topic	Item #	Checklist item	Location where item is reported
Selection process	8	Specify the methods used to decide whether a study met the inclusion criteria of the review, including how many reviewers screened each record and each report retrieved, whether they worked independently, and if applicable, details of automation tools used in the process.	**Subsection 2.2. Study selection, data extraction and risk of bias (in Section 2. Materials and Methods)** (page 3)
Data collection process	9	Specify the methods used to collect data from reports, including how many reviewers collected data from each report, whether they worked independently, any processes for obtaining or confirming data from study investigators, and if applicable, details of automation tools used in the process.	**Subsection 2.2. Study selection, data extraction and risk of bias (in Section 2. Materials and Methods)** (page 3)
Data items	10a	List and define all outcomes for which data were sought. Specify whether all results that were compatible with each outcome domain in each study were sought (e.g. for all measures, time points, analyses), and if not, the methods used to decide which results to collect.	**Subsection 2.3. Data collection: eligible trial design, participant characteristics and outcomes (in Section 2. Materials and Methods)** (pages 3–4)
	10b	List and define all other variables for which data were sought (e.g. participant and intervention characteristics, funding sources). Describe any assumptions made about any missing or unclear information.	**Subsection 2.3. Data collection: eligible trial design, participant characteristics and outcomes (in Section 2. Materials and**

PRISMA 2020 Checklist - The efficacy of glucagon-like peptide-1 based therapies in heart failure across the spectrum of left ventricular ejection fraction: a systematic review and meta-analysis *(Christophides T, et al)*

Table S1. PRISMA checklist			
**Section and Topic**	**Item #**	**Checklist item**	**Location where item is reported**
			**Methods)** (pages 3–4)
Study risk of bias assessment	11	Specify the methods used to assess risk of bias in the included studies, including details of the tool(s) used, how many reviewers assessed each study and whether they worked independently, and if applicable, details of automation tools used in the process.	**Subsection 2.2. Study selection, data extraction and risk of bias (in Section 2. Materials and Methods)** (page 3)
Effect measures	12	Specify for each outcome the effect measure(s) (e.g. risk ratio, mean difference) used in the synthesis or presentation of results.	**Subsection 2.4. Data analysis (in Section 2. Materials and Methods)** (page 4)
Synthesis methods	13a	Describe the processes used to decide which studies were eligible for each synthesis (e.g. tabulating the study intervention characteristics and comparing against the planned groups for each synthesis (item #5)).	**Subsections 2.1–2.3 (in Section 2. Materials and Methods)** (pages 3–4)
	13b	Describe any methods required to prepare the data for presentation or synthesis, such as handling of missing summary statistics, or data conversions.	**Subsections 2.1 and 2.2 (in Section 2. Materials and Methods)** (page 3)
	13c	Describe any methods used to tabulate or visually display results of individual studies and syntheses.	**Subsections 2.1 and 2.2 (in Section 2. Materials and Methods)** (page 3)
	13d	Describe any methods used to synthesize results and provide a rationale for the choice(s). If meta-analysis was performed, describe the model(s), method(s) to identify the presence and extent of statistical heterogeneity, and software package(s) used.	**Subsection 2.4. Data analysis (in**

Table S1. PRISMA checklist

Section and Topic	Item #	Checklist item	Location where item is reported
			**Section 2. Materials and Methods)** (page 4)
	13e	Describe any methods used to explore possible causes of heterogeneity among study results (e.g. subgroup analysis, meta-regression).	**Subsection 2.4. Data analysis (in Section 2. Materials and Methods)** (page 4)
	13f	Describe any sensitivity analyses conducted to assess robustness of the synthesized results.	**Subsection 2.4. Data analysis (in Section 2. Materials and Methods)** (page 4)
Reporting bias assessment	14	Describe any methods used to assess risk of bias due to missing results in a synthesis (arising from reporting biases).	**Subsection 2.2. Study selection, data extraction and risk of bias (in Section 2. Materials and Methods)** (page 3)
Certainty assessment	15	Describe any methods used to assess certainty (or confidence) in the body of evidence for an outcome.	**Subsection 2.4. Data analysis (in Section 2. Materials and Methods)** (page 4)
**RESULTS**			
Study selection	16a	Describe the results of the search and selection process, from the number of records identified in the search to the number of studies included in the review, ideally using a flow diagram.	**Subsection 3.1. Literature search and study**

Table S1. PRISMA checklist			
**Section and Topic**	**Item #**	**Checklist item**	**Location where item is reported**
			**selection (in Section 3. Results)** (page 4) **Figure S1** (page 5)
	16b	Cite studies that might appear to meet the inclusion criteria, but which were excluded, and explain why they were excluded.	**Subsection 3.1. Literature search and study selection (in Section 3. Results)** (page 4) **Figure S1** (page 5)
Study characteristics	17	Cite each included study and present its characteristics.	**Subsection 3.3. Study and patient characteristics (in Section 3. Results)** (page 5) **Supplementary Table S1 (Table S1)** (Supplementary Data)
Risk of bias in studies	18	Present assessments of risk of bias for each included study.	**Supplementary Figure S1 (Figure S1)** (Supplementary Data)
Results of individual studies	19	For all outcomes, present, for each study: (a) summary statistics for each group (where appropriate) and (b) an effect estimate and its precision (e.g. confidence/credible interval), ideally using structured tables or plots.	**Abstract** (page 1) **Section 3. Results and subsections 3.4–3.6, with**

Table S1. PRISMA checklist			
**Section and Topic**	**Item #**	**Checklist item**	**Location where item is reported**
			**relevant Figures (Figures 3–5)** (Pages 5–9)
Results of syntheses	20a	For each synthesis, briefly summarise the characteristics and risk of bias among contributing studies.	**Section 3. Results and subsections 3.4–3.6, with relevant Figures (Figures 3–5)** (Pages 5–9)<br><br>**Supplementary Figure S1 (Figure S1)** (Supplementary Data)<br><br>**Supplementary Figure S2 (Figure S2)** (Supplementary Data)
	20b	Present results of all statistical syntheses conducted. If meta-analysis was done, present for each the summary estimate and its precision (e.g. confidence/credible interval) and measures of statistical heterogeneity. If comparing groups, describe the direction of the effect.	**Section 3. Results and subsections 3.4–3.6, with relevant Figures (Figures 3–5)** (Pages 5–9)
	20c	Present results of all investigations of possible causes of heterogeneity among study results.	**Section 3. Results and subsections 3.4–3.6, with relevant Figures (Figures 3–5)** (Pages 5–9)
	20d	Present results of all sensitivity analyses conducted to assess the robustness of the synthesized results.	**Section 3.**

Table S1. PRISMA checklist			
**Section and Topic**	**Item #**	**Checklist item**	**Location where item is reported**
			**Results and subsections 3.4–3.6, with relevant Figures (Figures 3–5)** (Pages 5–9)
Reporting biases	21	Present assessments of risk of bias due to missing results (arising from reporting biases) for each synthesis assessed.	**Supplementary Figure S1 (Figure S1)** (Supplementary Data)
Certainty of evidence	22	Present assessments of certainty (or confidence) in the body of evidence for each outcome assessed.	**Section 3. Results and subsections 3.4–3.6, with relevant Figures (Figures 3–5)** (Pages 5–9)
**DISCUSSION**			
Discussion	23a	Provide a general interpretation of the results in the context of other evidence.	**Abstract** (page 1) **Section 4. Discussion** (page 10)
	23b	Discuss any limitations of the evidence included in the review.	**Section 4. Discussion** (page 13)
	23c	Discuss any limitations of the review processes used.	**Section 4. Discussion** (page 13)
	23d	Discuss implications of the results for practice, policy, and future research.	**Abstract** (page 1) **Section 5. Conclusions** (page 13)
**OTHER INFORMATION**			

PRISMA

PRISMA 2020 Checklist - The efficacy of glucagon-like peptide-1 based therapies in heart failure across the spectrum of left ventricular ejection fraction: a systematic review and meta-analysis *(Christophides T, et al)*

Table S1. PRISMA checklist

Section and Topic	Item #	Checklist item	Location where item is reported
Registration and protocol	24a	Provide registration information for the review, including register name and registration number, or state that the review was not registered.	**Abstract** (page 1) **Subsection 2.1. Search strategy and study identification (in Section 2. Materials and Methods)** (page 3)
	24b	Indicate where the review protocol can be accessed, or state that a protocol was not prepared.	**Abstract** (page 1) **Subsection 2.1. Search strategy and study identification (in Section 2. Materials and Methods)** (page 3)
	24c	Describe and explain any amendments to information provided at registration or in the protocol.	**Not applicable**
Support	25	Describe sources of financial or non-financial support for the review, and the role of the funders or sponsors in the review.	**Relevant section, Page 13**
Competing interests	26	Declare any competing interests of review authors.	**Relevant section, Page 13**
Availability of data, code and other materials	27	Report which of the following are publicly available and where they can be found: template data collection forms; data extracted from included studies; data used for all analyses; analytic code; any other materials used in the review.	**Relevant section, Page 13**

From: Page MJ, McKenzie JE, Bossuyt PM, Boutron I, Hoffmann TC, Mulrow CD, et al. The PRISMA 2020 statement: an updated guideline for reporting systematic reviews. BMJ 2021;372:n71. doi: 10.1136/bmj.n71.
This work is licensed under CC BY 4.0. To view a copy of this license, visit https://creativecommons.org/licenses/by/4.0/

Table S2. Characteristics of studies used in the meta-analysis [7,13–25]

	**Pfeffer *et al*, 2015**	**Kyhl *et al*, 2016**	**Margulies *et al*, 2016**	**Chen *et al*, 2016**

PRISMA 2020 Checklist - The efficacy of glucagon-like peptide-1 based therapies in heart failure across the spectrum of left ventricular ejection fraction: a systematic review and meta-analysis *(Christophides T, et al)*

	(ELIXA)		(FIGHT)	
**Study duration**	2010–2013	2009–2025	2013–2015	2013–2016
**DOI**	10.1056/NEJMoa1509225	10.4244/EIJV12I4A78	10.1001/jama.2016.10260	10.1007/S12020-015-0798-0
**Location**	NA	Denmark	USA	China
**Centre**	Multicentre	Multicentre	Multicentre	Single
**Nationality**	Multinational	Single	Multistate	Single
**Study type**	Randomised, blinded, placebo-control	Randomised, blinded, placebo-control	Randomised, blinded, placebo-control	Randomised, blinded, placebo-control
**GLP-1 agent**	Lixisenatide	Exenatide	Liraglutide	Liraglutide
**HF group, *n***	1358[a]	334	300	90
**Males, *n***	ND	264	239	66
**Female, *n***	ND	70	61	24
**Rx/Comparison, *n***	682/676	175/159	154/146	45/45
**Completed, *n***	NA	NA	271	83
**Male completed, *n***	NA	NA	207	ND
**Female completed, *n***	NA	NA	64	ND
**Rx/Comparison, *n***	NA / NA	NA / NA	106/103	41/42
**Age, years - all groups**<br>**Rx/Comparison**	ND<br>ND / ND	ND<br>62 (±11)/63 (±12) [SD]	61 (52, 68)<br>62 (52, 68)/61 (51, 67)	61 (52, 68)<br>58 (±11.7)/59 (±12.1) [SD][c]
**Follow-up**	24 weeks	5.2 years[b]	180 days	6 months
**DM pt proportion**	100%	9%	59%	24%
**CAD pt proportion**<br>**(ACS pt)**	100%<br>(yes)	100%<br>(yes)	82%<br>(no)	100%<br>(yes)
**BMI - all groups**<br>**Rx/Comparison**	ND<br>ND / ND	ND<br>27 (±4)/24 (±4) [SD]	ND<br>31 (26, 36)/33 (25, 38)	ND<br>25.2 (±3.4)/25 (±3.1) [SD]
**Target HF subgroup**	ND	ND	HFrEF	ND[d]
**NYHA classes**	ND	ND	II(29%)-III(63%)-IV(5%)	ND
**HF prognostic drugs**	ND	11% ACEi/ARB, 7% BB, ND MRA (NA for ARNI-ARB and SGLG2-i)	72% ACEi/ARB, 94% BB, 59% MRA (NA for ARNI-ARB and SGLG2-i)	51% ACEi/ARB, 90% BB, 8% MRA (NA for ARNI-ARB and SGLG2-i)
**LVEF % - all groups**<br>**Rx/Comparison**	25 (19, 32)<br>ND/ND	ND<br>47 (±10)/48 (±9) [SD]	25 (19, 33)<br>25 (20, 33)/25 (19, 32)	ND<br>47.2 (±5.1)/47.7 (±5.1) [SD]

Data shown are mean (± standard deviation (SD) or standard error of the mean (SEM)) or median (with interquartile range (IQR)). [a]Subgroup analysis of ELIXA trial focusing on heart failure (HF) patients. Total *n* refers to subgroup with HF. [b]Overall study follow-up was 5.2 years, with the exception of the left ventricular ejection fraction (LVEF) change, which was reported at 90 days. [c]Age reported as a combination of median and means. [d]Study included all acute coronary syndrome patients (ACS pt) without specific LVEF target at baseline. Additional abbreviations: ACE-i, angiotensin converting enzyme inhibitor; ARB, angiotensin receptor blocker; ARNI, angiotensin receptor neprilysin inhibitor; BB, beta blocker; BMI, body mass index; CAD pt, coronary artery disease patients; DM pt, diabetes mellitus patients; GLP-1, glucagon-like peptide-1; HFrEF, heart failure with reduced ejection fraction; MRA, mineralocorticoid receptor antagonist; NA, not applicable; ND, not defined; NYHA, New York Heart Association; Rx, treatment group; SGLG2-i, sodium-glucose cotransporter-2 inhibitors.

Table S2 (continued). Characteristics of studies used in the meta-analysis [7,13–25]

	**Marso *et al*, 2020 (LEADER)**	**Branch *et al*, 2022 (REWIND)**	**Ferreira *et al*, 2022 (HARMONY OUTCOMES)**	**Packer *et al*, 2024 (SUMMIT)**
**Study duration**	2010–2015	2011–2018	2015–2018	2021–2024
**DOI**	10.1016/J.JACC.2019.12.063	10.1002/EJHF.2670	10.1002/EJHF.2660	10.1056/NEJMoa2410027
**Location**	NA	NA	NA	NA
**Centre**	Multicentre	Multicentre	Multicentre	Multicentre
**Nationality**	Multinational	Multinational	Multinational	Multinational
**Study type**	Randomised, blinded, placebo-control	Randomised, blinded, placebo-control	Randomised, blinded, placebo-control	Randomised, blinded, placebo-controlled
**GLP-1 agent**	Liraglutide	Dulaglutide	Albiglutide	Tirzepatide
**HF group, *n***	1667	853	1922	731
**Males, *n***	983	488	1251	338
**Female, *n***	684	365	671	393
**Rx/Comparison, *n***	835 / 832	421 / 432	952 / 966	364/367
**Completed, *n***	NA	NA	NA	ND
**Male completed, *n***	NA	NA	NA	ND
**Female completed, *n***	NA	NA	NA	ND
**Rx/Comparison, *n***	NA/NA	NA/NA	NA/NA	ND/ND
**Age, years - all groups**<br>**Rx/Comparison**	63.7 (±7.8)<br>63.5 (±7.8)/64 (±7.8) [SD]	65.7 (±7.4) [SD]<br>ND/ND	65 (58, 70)<br>ND/ND	65.2[f]<br>65.5 (±10.5)/65 (±10.9) [SD]
**Follow-up**	5 years	5.4 years[c]	1.6 years[e]	104 weeks
**DM pt proportion**	100%	100%	100%	48%
**CAD pt proportion**<br>**(ACS pt)**	ND<br>(NA)	36%<br>(no)	81%<br>(no)	30%<br>(no)
**BMI - all groups**<br>**Rx/Comparison**	34 (±6.9)<br>34.2 (±6.9)/33.9 (±6.8)	33.8 (±6.3)<br>ND/ND	32.6 (29, 37)<br>ND/ND	ND<br>38.3(±6.4)/38.2(±7) [SD]
**Target HF subgroup**	ND	ND[d]	ND	HFpEF
**NYHA classes**	I(21%)-II(65%)-III(13%)[a]	ND	I/II(87%)-III(ND)-IV(ND)	II(73%)-III/IV(27%)
**HF prognostic drugs**	87% ACEi/ARB, 73% BB, 16% MRA, ND ARNI-ARB, 0.2 – 0.3% SGLG2-i[b]	84% ACEi/ARB, 78% BB, 26% MRA, ND ARNI-ARB, 0.1% SGLG2-i	86% ACEi/ARB, 82% BB, 21% MRA, ND ARNI-ARB, 5% SGLG2-i	81% ACEi/ARB/ARNI, 69% BB, 35% MRA, 17% SGLG2-i
**LVEF % - all groups**<br>**Rx/Comparison**	ND<br>ND/ND	ND<br>ND/ND	ND<br>ND/ND	ND<br>61 (±6.5)/60.6 (±6.2) [SD]

Data shown are mean (± standard deviation (SD) or standard error of the mean (SEM)) or median (with interquartile range (IQR)). [a]0.8% of trialists had unknown New York Heart Association (NYHA) status. [b]Sodium-glucose cotransporter-2 inhibitor (SGLG2-i) use was reported for treatment (Rx) and placebo groups (0.2% and 0.3% respectively) but not for the overall group. [c]Follow-up reported as a median. [d]No definition of left ventricular ejection fraction (LVEF) and no prespecified stratification by heart failure (HF) status. [e]Follow-up reported as a median. [f]Overall age given as mean without SD/SEM reported. Additional abbreviations: ACE-i, angiotensin converting enzyme inhibitor; ACS pt, acute coronary syndrome patients; ARB, angiotensin receptor blocker; ARNI, angiotensin receptor neprilysin inhibitor; BB, beta blocker; BMI, body mass index; CAD pt, coronary artery disease patients; DM pt, diabetes mellitus patients; GLP-1, glucagon-like peptide-1; HFpEF, heart failure with preserved ejection fraction; MRA, mineralocorticoid receptor antagonist; NA, not applicable; ND, not defined.

Table S2 (continued). Characteristics of studies used in the meta-analysis [7,13–25]

	**Butler *et al*, 2024[a] (STEP-HFpEF)**	**Neves *et al*, 2024 (EXSCEL)**	**Kosiborod *et al*, 2024 (STEP-HFpEF DM)**
**Study duration**	2021–2023	2010–2017	2021–2023

**DOI**	10.1016/j.cardfail.2023.10.465	10.1002/ejhf.3478	10.1056/nejmoa2313917
**Location**	NA	NA	NA
**Centre**	Multicentre	Multicentre	Multicentre
**Nationality**	Multinational	Multinational	Multinational
**Study type**	Randomised, blinded, placebo-control	Randomised, blinded, placebo-controlled	Randomised, blinded, placebo-controlled
**GLP-1 agent**	Semaglutide	Exenatide	Semaglutide
**HF group, *n***	529 (85 for 45-49%, 215 for 50-59%, 229 for ≥ 60%)	455	616
**Males, *n***	232 (55 for 45-49%, 96 for 50-59%, 81 for ≥ 60%)	366	343
**Female, *n***	297 (30 for 45-49%, 119 for 50-59%, 148 for ≥ 60%)	89	273
**Rx/Comparison, *n***	37 / 48 for 45-49%, 113 / 102 for 50-59%, 113 / 116 for ≥ 60%	249/206	310/306
**Completed, *n***	NA	NA	583
**Male completed, *n***	NA	NA	ND
**Female completed, *n***	NA	NA	ND
**Rx/Comparison, *n***	NA/NA	NA/NA	292/291
**Age, years - all groups**<br>**Rx/Comparison**	69 (59, 74) for 45-49%, 69 (63, 76) for 50-59%, 70 (62, 75) for ≥ 60%<br>ND/ND (for all groups)	ND<br>61.2/62.9[b]	69<br>69 (62, 74)/70 (63, 75)
**Follow-up**	52 weeks	3.2 years	52 weeks
**DM pt proportion**	0% for all groups	100%	100%
**CAD pt proportion**<br>**(ACS patients)**	41%<br>(no)	91%<br>(no)	24%<br>(no)
**BMI - all groups**<br>**Rx/Comparison**	36 (33.9, 39.8)<br>ND/ND (for all groups)	ND<br>30.5/29.5[c]	ND[c]<br>36.9 (33.6, 41.5)/36.9 (33.5, 41.1)
**Target HF subgroup**	HFmrEF, HFpEF	HFrEF[d]	HFpEF
**NYHA proportions**	II(65%)-III/IV(35%) for 45-49%, I(68%)-III/IV(32%) for 50-59%, II(65%)-III/IV(35%) for ≥ 60%	I(18.5%)-II(56.5%)-III(22%)-IV(3%)	II(71%)-III/IV(29%)
**HF prognostic drugs**	72% ACEi/ARB, 78% BB, 29% MRA , 4% ARNI-ARB, 3% SGLG2-i for ≥ 60%	89% ACEi/ARB, 88% BB, 35% MRA, ND ARNI-ARB, 0.2% SGLG2-i	81% ACEi/ARB/ARNI, 83% BB, 32%MRA, 33% SGLG2-i
**LVEF % - all groups**<br>**Rx/Comparison**	46 (45, 48) for 45-49%, 55 (52, 56) for 50-59%, 60 (60, 65) for ≥ 60%<br>ND/ND (for all groups)	LVEF<40%[d]<br>ND/ND	ND<br>57 (50, 61)/55 (50, 60)

Data shown are mean (± standard deviation (SD) or standard error of the mean (SEM)) or median (with interquartile range (IQR)). [a]STEP-HFpEF trial post-hoc analysis with 3 heart failure (HF) groups based on left ventricular ejection fraction (LVEF), (1 group refers to the HF with mildly reduced ejection fraction (HFmrEF) category (45-49% LVEF), and 2 groups fall into the HF with preserved ejection fraction (HFpEF) category (50-59% and ≥60% LVEF). [b]Age given as mean without SD/SEM reported. [c]Body mass index (BMI) given as median without IQR. [d]Outcomes primarily reported for LVEF <40% or for HF with reduced ejection fraction (HFrEF), though limited outcomes (with limited baseline data) for LVEF 40-49% (or HFmrEF) and >55% (or HFpEF) were also available. Additional abbreviations: ACE-i, angiotensin converting enzyme inhibitor; ACS pt, acute coronary syndrome patients; ARB, angiotensin receptor blocker; ARNI, angiotensin receptor neprilysin inhibitor; BB, beta blocker; CAD pt, coronary artery disease patients; DM pt, diabetes mellitus patients; GLP-1, glucagon-like peptide-1; MRA, mineralocorticoid receptor antagonist; NA, not applicable; ND, not defined; Rx, treatment group; SGLG2-i, sodium-glucose cotransporter-2 inhibitors; NYHA, New York Heart Association.

Table S2 (continued). Characteristics of studies used in the meta-analysis [7,13–25]

	**Pratley *et al*, 2024 (FLOW)[a]**	**Deanfield *et al*, 2024 (SELECT)**	**Pop-Busui *et al*, 2025 (SOUL)**
**Study duration**	2019–2024	2018–2023	2019–2021
**DOI**	10.1016/j.jacc.2024.08.004	10.1016/S0140-6736(24)01498-3	10.1093/eurheartj/ehaf784.4317
**Location**	NA	NA	NA
**Centre**	Multicentre	Multicentre	Multicentre

**Nationality**	Multinational	Multinational	Multinational
**Study type**	Randomised, blinded, placebo-controlled	Randomised, blinded, placebo-controlled	Randomised, blinded, placebo-controlled
**GLP-1 agent**	Semaglutide	Semaglutide	Semaglutide
**HF group, *n***	678	1347 for HFrEF, 2273 for HFpEF	592 for HFrEF, 991 for HFpEF
**Males, *n***	431	1147 for HFrEF, 1567 for HFpEF	ND
**Female, *n***	247	200 for HFrEF, 706 for HFpEF	ND
**Rx/Comparison, *n***	342/336	654/693 for HFrEF, 1174/1099 for HFpEF	296/296 for HFrEF, 494/497 for HFpEF
**Completed, *n***	NA	NA	NA
**Male completed, *n***	NA	NA	NA
**Female completed, *n***	NA	NA	NA
**Rx/Comparison, *n***	NA/NA	NA/NA	NA/NA
**Age, years - all groups**<br>**Rx/Comparison**	68 (63, 73)<br>ND/ND	61.7 (±8.7) [SD]<br>ND/ND	66.1 (±7.6) [SD]<br>ND/ND
**Follow-up**	3.4 years[b]	39.8 months[c]	47.5 months[c]
**DM pt proportion**	100%	0%	100%
**CAD pt proportion**<br>**(ACS patients)**	75%<br>(no)	79% for HFrEF, 70% for HFpEF<br>(no)	ND<br>(NA)
**BMI - all groups**<br>**Rx/Comparison**	33.3 (29.8, 38)<br>ND/ND	33.4 (±4.9) for HFrEF, 34 (±5.4) for HFpEF [SD]<br>ND/ND	31.8 (±ND) [SD]<br>ND/ND
**Target HF subgroup**	HFrEF, HFmrEF, HFpEF	HFrEF, HFpEF	HFrEF, HFpEF
**NYHA classes**	I(32%)-II(57%)-III(11%)	I(28%)-II(62%)-III(10%) for HFrEF, I(33%)-II(59%)-III(8%) for HFpEF	ND
**HF prognostic drugs**	95% ACEi/ARB, 77% BB, 17% MRA, 0.3% ARNI-ARB, 12% SGLG2-i	83% ACEi/ARB, 90% BB, 48% MRA, 13% ARNI-ARB, ND SGLG2-i for HFrEF<br>83% ACEi/ARB, 82% BB, 18% MRA, 1% ARNI-ARB, ND SGLG2-i for HFpEF	30% SGLG2-I for HFrEF and HFpEF, other drugs ND
**LVEF % - all groups**<br>**Rx/Comparison**	(3 subgroups, LVEF<40%, 40-49%, ≥50%)<br>ND/ND	(2 main subgroups, LVEF<40%, LVEF ≥50%)[d]	ND<br>(NA/NA)

Data shown are mean (± standard deviation (SD) or standard error of the mean (SEM)) or median (with interquartile range (IQR)). [a]In our extraction we analysed data 'by left ventricular ejection fraction (LVEF)' - thus *n* used is different for each outcome and reflects participants with that LVEF whose outcomes were assessed (heart failure (HF) with: reduced ejection fraction (HFrEF), *n* = 308; mildly reduced ejection fraction (HFmrEF), *n* = 66; preserved ejection fraction (HFpEF), *n* = 69). The HF subgroup had 336 placebo and 342 Rx participants (*n* for specific subgroups not available). Overall HF population values used for New York Heart Association (NYHA) classification, age, prognostic drugs. [b]Follow-up reported as a median. [c]Follow-up reported as a mean. [d]Outcomes generally divided into HFpEF and HFrEF groups. Patients with HFmrEF (LVEF 40-49%) appear in both HFpEF and HFrEF subgroups (10% in HFpEF subgroup have HFmrEF and 46% in HFrEF subgroup have HFmrEF). Additional abbreviations: ACE-i, angiotensin converting enzyme inhibitor; ACS pt, acute coronary syndrome patients; ARB, angiotensin receptor blocker; ARNI, angiotensin receptor neprilysin inhibitor; BB, beta blocker; BMI, body mass index; CAD pt, coronary artery disease patients; DM pt, diabetes mellitus patients; GLP-1, glucagon-like peptide-1; MRA, mineralocorticoid receptor antagonist; NA, not applicable; ND, not defined; Rx, treatment group; SGLG2-i, sodium-glucose cotransporter-2 inhibitors.

PRISMA 2020 Checklist - The efficacy of glucagon-like peptide-1 based therapies in heart failure across the spectrum of left ventricular ejection fraction: a systematic review and meta-analysis *(Christophides T, et al)*

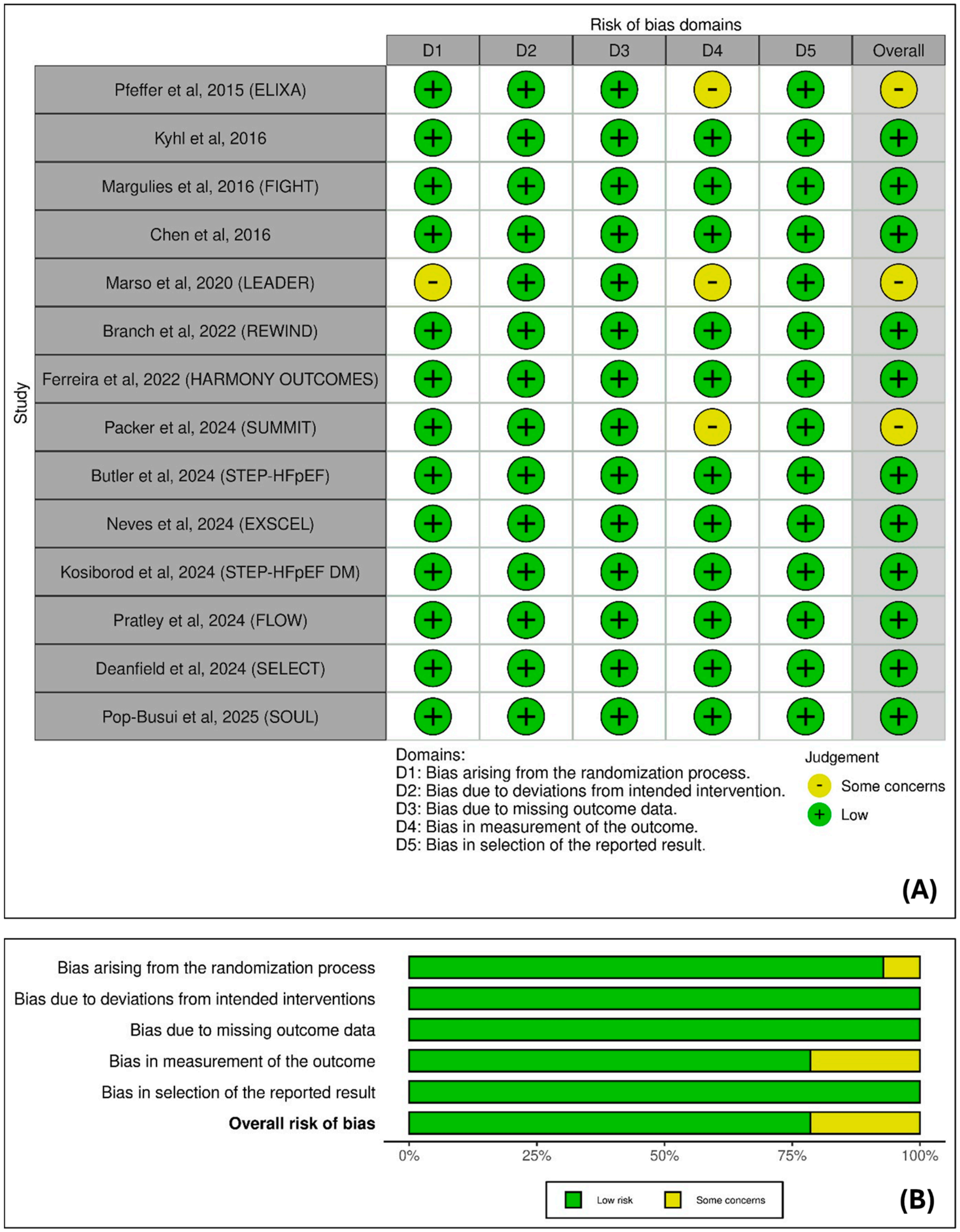

Figure S1. Risk of bias assessment for all included studies using the revised Cochrane assessment tool: **(A)** traffic light plot for individual studies and **(B)** summary plot [7,13–25].

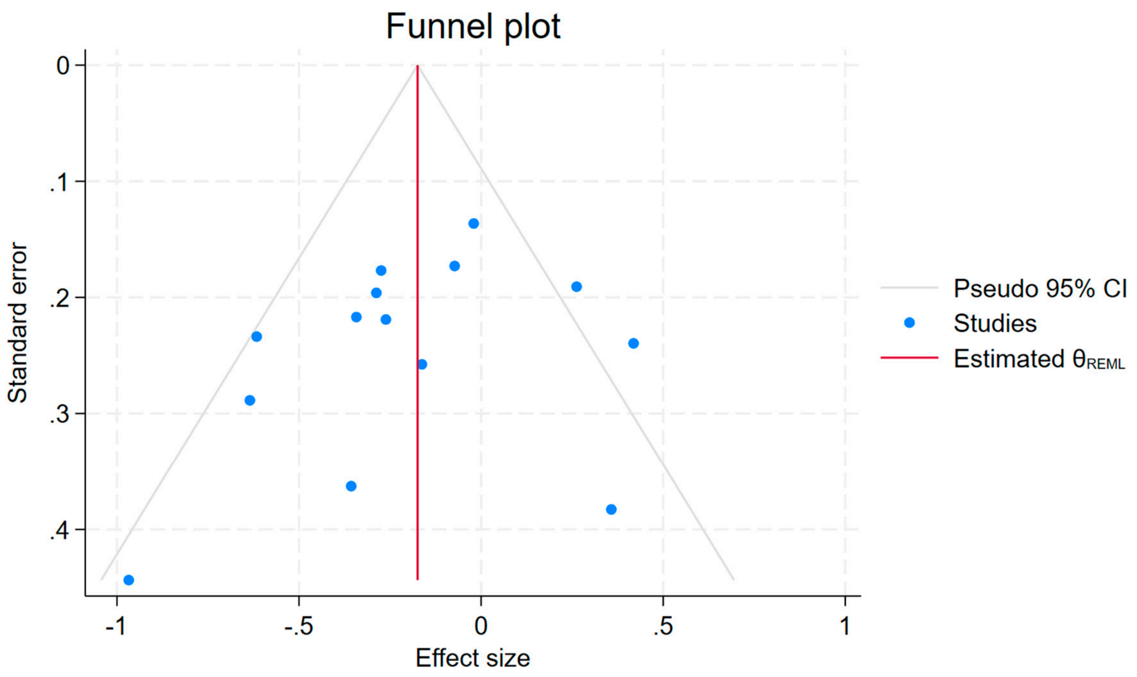

Figure S2. Publication bias assessment - funnel plot for heart failure hospitalisations [7,13,15–20,22,23].

Abbreviations: CI, confidence interval; REML, restricted maximum likelihood.
